# Primary central nervous system plasmablastic lymphoma presenting in human immunodeficiency virus-negative but Epstein-Barr virus-positive patient: A case report

**DOI:** 10.1186/1746-1596-7-51

**Published:** 2012-05-08

**Authors:** Li Ying Zhang, Hui Yun Lin, Lan Xiang Gao, Lin Li, Yu Wang Tian, Zhi Qin Liu, Xiao Hua Shi, Zhi Yong Liang

**Affiliations:** 1 Department of Pathology, The Military General Hospital of Beijing PLA, Members of Chinese Medical Association, Nanmen Warehouse 5, Dongsishitiao Street, Dongcheng District, Beijing, 100700, People's Republic of China; 2 Department of Image, The Military General Hospital of Beijing PLA, Beijing, 100700, People's Republic of China; 3 Department of Pathology, Peking Union Medical College Hospital, Beijing, 100032, People's Republic of China

**Keywords:** Plasmablastic lymphoma, Central nervous system, Human immunodeficiency virus, Epstein-barr virus

## Abstract

**Abstract:**

We report a 32-year-old Outer Mongolian man, with plasmablastic lymphoma (PBL) primarily occured in the central nervous system and diagnosed by surgical resection. This patient appeared headache and Magnetic resonance imaging (MRI) showed multiple lesions in the right cerebral hemisphere including the right frontal-parietal lobe and right basal ganglia and the left cerebellum, he was diagnosed as lymphoma by stereotactic biopsy in January 2009 in local hospital, and was given radiotherapy 33 times after the biopsy. The patient was admitted to The Military General Hospital of Beijing PLA., Beijing, P.R. China on March 9th, 2011, with chief complaints of right limbs convulsioned suddenly, then fell down and lose of his consciousness, then awoke after 4 to 5 minutes, with symptoms of angulus oris numbness and the right upper limb powerless ten days ago.

MRI of the brain revealed a well-defined hyperdense and enhancing mass in the left frontal-parietal lobe, the meninges are closely related, there was extensive peritumoural edema noted with pressure effects, as evident by effacement of the left lateral ventricles and a 0.5 cm shift of the midline to the right side.

Surgical resection showed markedly atypical, large singly dispersed or cohesive proliferation of plasmacytoid cells with frequent abnormal mitoses and binucleation, some neoplastic cells were large with round or oval nuclei and showed coarse chromatin and smaller or unapparent nucleoli, some neoplastic cells with prominent nucleoli, apoptosis and necrosis were often presented. Immunohistochemistry staining and gene rearrangement together with other supportive investigation confirmed the diagnosis of primary central nervous system plasmablastic lymphoma. A month later, he was started on chemotherapy with R-CHOP (rituximab, cyclophosphamide, doxorubicin, leurocristime and prednisone) for a week. Other supportive treatment was provided for symptomatic epilepsy. The patient regained muscle strength in both upper limbs and right lower limb and the symptomatic epilepsy was controlled after two weeks. Then the patient was discharged. Follow-up data shows the patient to be alive eleven months after discharge.

**Virtual Slides:**

The virtual slide(s) for this article can be found here: http://www.diagnosticpathology.diagnomx.eu/vs/1649317674697046.

## Background

Unlike the numerous subtypes of extra-central nervous system (CNS) lymphoma, most primary central nervous system lymphomas (PCNSLs) are a high-grade non-Hodgkin’s subtype with the features of diffuse large B cell lymphoma (DLBCL). So far there were two cases of primary central neurous system PBL reports in the world. One case occurred in the right basal ganglian [1], another occurred in the left anterior frontal lobe [2]and both patients were HIV-positive. Here we report a HIV-negative but EBV-positive patient with a primary CNS plasmablastic lymphoma, a rare variant of DLBCL.

## Case presentation

A 32-year-old Outer Mongolian man, who is a freelancer, was born and grew up in the city of Ulan Bator, Capital of Outer Mongolia. Ten days ago his right limbs convulsioned suddenly, then he fell down and lose of consciousness, and awoke after 4 to 5 minutes, with symptoms of angulus oris numbness and the right upper limb powerless. He was referred to The Military General Hospital of Beijing PLA., Beijing, P.R. China on March 9th, 2011.

The patient accepted a eye operation because of acute glaucoma in 2008, he denied any infection and family histories such as HIV, HBV, HCV, HPV, Syphilis infections, cancer or hereditary diseases or organ transplantation history. He never knew the infection of EBV before this admission. He had no bad habits such as smoking and alcoholism, no dust, toxins, radioactive material exposure history, had never been to infectious disease areas, had no habit of eating raw fish and raw meat, etc.

**Figure 1:**
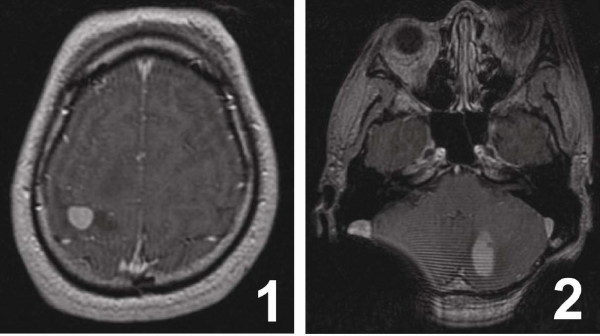
**MR imaging of the head in 2009.(1)** The MRI T1 Weighted Imaging shows that there are multiple masses with high signals in the margin and low signals in the center, and T2 Weighted Imaging shows equal signals, in the right cerebral hemisphere, especially in the right frontal-parietal lobe and the basal ganglia area, and edema with low signals could be seen around the mass. **(2)** The left cerebellum is also visible of abnormal signals.

**Figure 2:**
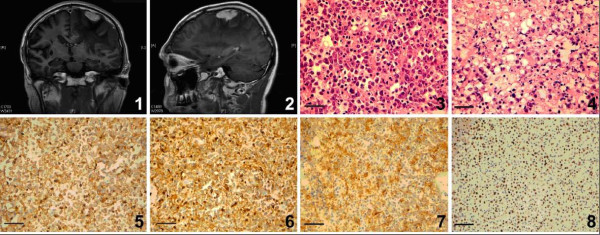
**1, 2 MR imaging of the head in 2011.** The MRI T1Weighted Imaging shows that there is a irregular mass with equal T2 and a little bit long T1 signals in the left frontal-parietal lobe, which is close to the outer edge of the meninges and a large finger-like edema around the mass with the proof of the left lateral ventricle is pressed significantly and a 0.5 cm shift of the midline to the right side. **(1)** coronal plane; **(2)** sagittalia plane. **3, 4 Hymatoxylin and eosin (H&E) shows the morphologic features of the neoplastic cells.** The neoplastic cells are large, round or oval, either diffused or sheet-spooty distributed, the cytoplasm is weak pink staining, some neoplastic cells like plasmatic cells with nuclei to one side, and some nuclei looks like “clock face” shape. Some cells shows coarse chromatin with smaller or unapparent nucleoli, but some cells have apparent nucleoli. The pathological mitosis and apoptosis are easy to find. (Magnification, ×400). **5**–**7 Immunohistochemistry staining shows the expression of LCA, CD38, CD79a in the tumor tissues.** Positive IHC signals are visualized with brown yellow color **(5)** There are diffused LCA positive signals in the cytoplasm of a great number of neoplastic cells. **(6)** Extensive CD38 staining are detectable in numerous neoplastic cells. (5) Some neoplastic cells are identified using CD79a IHC staining. (Magnification, ×200). **8*In situ*hybridization shows EBV EBER expression in the tumor tissues.** Positive ISH signals are visualized with brown yellow color. There are significant positive siganals in most of the neoplastic cells. (Magnification, ×200).

Two years prior to this hospital admission, this patient began no incentive headache in January 2009, the headache progressively serious, with some symptoms such as hiccups, nausea, vomiting, urgent urination, dry stool, and the left limb weakness which resulting in unsteady gait. MRI inspection in local hospital showed multiple lesions in the right hemisphere, especially in the right frontal-parietal lobe and basal ganglia and the left cerebellum areas (Figure 1, 2). The patients carried the imaging films on his admission which were done in local hospital. He dictated that he was dignosed as lymphoma with biopsy in local hospital, but the inspection of the neoplastic tissues was too small to make a final and exact diagonosis, he was given radiotherapy 33 times after the operation, the exact radiation regimens and doses were whole skull (DT39.6 Gy/22f) and left cerebellum (DT19.8 Gy/11f), with slight side effects such as hair loss and sometimes uausea during the radiotherapy. He felt intermittent headache but never carry out any examination and treatment before this time admission.

On admission, the patient was with no fever, papilloedema, but some neurological deficits including the myodynamia of the proximal end of right upper limb was stage IV, distal end of the right upper limb was stage III, the myodynamia of the right lower limb was stage IV, and Babinski syndrome (+), etc. noted on physical examination. Physical inspection revealed no enlargement of general superficial lymph nodes and no mass was found in the head and neck region, including the oral cavity.

Blood inspection showed that WBC 7.63x10^9^/L, neutrophils 80.9%, RBC 4.6x10^12^/L, hemoglobin 139.0 g/L, PLT 234x10^9^/L; Biochemical inspection showed that ALT 17 U/L, TBIL 9.60umol/L, DBIL 4.1 umol/L, TP 60.3 g/L, ALB 46.2 g/L, GLU 6.4 mmol/L, BUN 5.90 mmol/L, Cr 67.0 umol/L, all these indexes were within the normal range. He was detected with no HIV, HBsAg, HBcAg, HPV, Syphilis infection after this admission.

MRI of the head revealed a well-defined hyperdense and enhancing mass, measuring 3.6 cm × 3.0 cm × 1.5 cm, in the left frontal-parietal lobe (Figure 2 1, 2). The lateral border of the mass was closely related to the meninges. There was extensive peritumoural edema noted with pressure effects, as evident by effacement of the left lateral ventricles and a 0.5 cm shift of the midline to the right side. PET/CT revealed no enlargement of deep lymph nodes and no mass in the head and neck region, including the oral cavity, and in other regions of the peripheral nervous system or organs.

**Figure 3:**
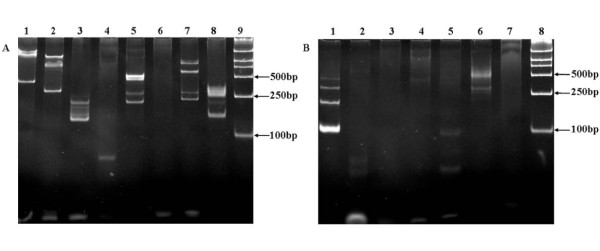
**Gene arrangement assays of BCR (IgH, IgK, IgL) and TCR in the tumor tissues.** Positive gene arrangements of BCR (IgH, IgK and IgL) could be detected in the tumor tissues, but TCR β, δ and γ could not be. **a. BCR (IgH, IgK, IgL) gene arrangement assays.** Lane 1. IgH: VH-FR1 + JH consensus (+); Lane 2, IgH: VH-FR2+ JH consensus (+); Lane 3, IgH: VH-FR3+ JH consensus (+); Lane 4, DH + JH consensus (−); Lane 5, DH7+ consensus (+); Lane 6, IgK: Vκ + Jκ (−); Lane 7, IgK: Vκ and intron + Kde (+); Lane 8, IgL: Vκ + Jλ (+); Lane 9, Marker. **b. TCR gene arrangement assays.** Lane 1, DNA ladder; Lane 2, TCRβ: Vβ + Jβ1/2 (−); Lane 3, TCRβ: Vβ + Jβ2 (−); Lane 4, TCRβ: Dβ + Jβ1/2 (−); Lane 5, TCRδ: Vδ + Dδ + Jδ (−); Lane 6. TCRγ: Vγ1-8, Vγ10 + multiple Jγ regions (−); Lane 7, TCRγ: Vγ9, Vγ11 + multiple Jγ regions (−); Lane 8, Marker.

Haematoxylin & eosin stain showed markedly atypical, large singly dispersed or cohesive proliferation of plasmacytoid cells with frequent abnormal mitoses and binucleation, some neoplastic cells were large with round or oval nuclei and showed coarse chromatin and smaller or unapparent nucleoli, some neoplastic cells with prominent nucleoli, apoptosis and necrosis were often presented (Figures 2 3, 4), no brain tissue was found in the specimen under the microscope. Immunohistochemstry staining showed that the neoplastic cells were positive for LCA (Figure 2 5), CD38 (Figure 2 6), CD79a (Figure 2 7), Plasma cell marker, Mum-1, kappa and lambda light chains partly positive, CD20 weakly positive, and negative for CD2, CD3, CD4, CD5, CD7, CD8, CD56, CD30, CD138, ALK, TIA-1 and PAX5. Ki-67 positive index was about 75% (all antibodies 1:100, Santa Cruz, Biotechnology, Int., CA) (Table 1). EBER *in situ* hybridization on a paraffin-embedded sections revealed the infection of nearly all plasmablastic lymphoma cells by Epstein-Barr virus (EBER probes, PanPath, Holand) (Figure 2 8). Gene rearrangement assays followed the protocols of the Biomed-2 PCR kit (InVivoScribe, CA), and the results showed that IgH, IgK and IgL were positive, but TCRβ, TCRδ and TCRγ were totally negative. (TCRβ [Vβ + Jβ1/2(−),Vβ + Jβ2(−),Dβ + Jβ1/2(−)]; TCRδ [Vδ + Dδ + Jδ(-)]; TCRγ [Vγ1-8,Vγ10 + multiple Jγregions (−); Vγ9, Vγ11+ multiple Jγregions (−)]; IgH [VH-FR1 + JH Consensus (+); VH-FR2 + JH Consensus (+); VH-FR3 + JH Consensus (+); DH + JH Consensus (−); DH7 + JH Consensus (+)]; IgK [Vκ + Jκ (−); Vκand intron + Kde (+)]; IgL [Vλ + Jλ(+)].) (Figure 3).

**Table 1:** Details of the results of immunohistochemistry staining in tumor cells

**Antibody**	**Result**	**Antibody**	**Result**
LCA	Diffusely cell membrane positive	CD4	Negative
CD38	Diffusely cell membrane positive	CD5	Negative
CD79a	Diffusely cell membrane positive	CD7	Negative
Mum-1	Partly nuclei positive	CD8	Negative
Kappa light chain	Partly cytoplasm positive	CD56	Negative
Lambda light chain	Partly cytoplasm positive	CD30	Negative
CD20	Partly cell membrane positive	CD138	Negative
Plasma cell marker	Partly cytoplasm positive	ALK	Negative
Ki-67	Nuclei positive index was about 75%	TIA-1	Negative
CD2	Negative	PAX5	Negative
CD3	Negative		

Follow up data by regular visits to this patient, after the operation, the patient was given anti-epileptic therapy, and started on chemotherapy with R-CHOP (rituximab, cyclophosphamide, doxorubicin, leurocristime and prednisone) for one week. Two weeks later, the symptoms of right limbs twitching were well controled, hemiplegy of right limbs mainly disappeared. Then the patient was discharged. Follow-up data shows the patient to be alive eleven months after discharge.

## Conclusions

Plasmablastic lymphoma (PBL) is a rare, highly invasive lymphoma, with diffuse proliferation of large neoplastic cells most of which resemble B immunoblasts, some tumor cells have immunophenotype of plasma cells. It was a unique subtype of diffuse large B cell lymphoma. It usually occures in HIV-positive individuals, predominantly males. In recent years more researches show that the pathogenesis of PBL has relationship with EBV and HHV8 [3-5]. Tereza CC *et al.* revealed that the frontal cortex is the main region to be frequently observed neurological lesions after being infected by Bovine Herpesvirus type 5 (BoHV-5) comparing with parietal cortex, thalamus and mesencephalon [6]. To the best of our knowledge, this is the first case report for PBL occurence in the intracranial frontal and parietal cortex region with HIV negative but EBV positive, demonstrating the occurance of this disease had relationship with the infection of EBV.

In this case immunophenotype showed that the neoplastic cells express a plasma cell phenotype including CD38, positive for LCA, CD79a and Mum-1, high Ki67 proliferation index, extronodal localization, and the presence of EBV by *in situ* hybridization for EBER all supported the diagnosis of plasmablastic lymphoma.

In PBL clonal IgH chain and MYC gene rearrangement is demonstrable, especially in those EBV-positive patients, may show evidence of somatic hypermutation or be in an unmutated configuration [7]. In this case the plasmacytoid neoplastic cells occurred IgH gene rearrangement, both kappa and lambda light chains by immunohistochemistry staining and gene rearrangement using polymerase chain reaction method, it illustrated that this case was a neoplastic lesions.

PBL should be morphologically distinguished with anaplastic or plasmablastic plasma cell myeloma, immunoblastic cell type diffuse large B-cell lymphoma, anaplastic diffuse large B-cell lymphoma, ALK-positive large B-cell lymphoma, primary effusion lymphoma and HHV8-related origin of Castleman's disease, multi-center B-cell lymphoma, and so on. It is easy to make the correct diagnosis according to clinical history (immune deficiency), disease site, tumor cell phenotype, high proliferation index and EBER *in situ* hybridization, etc. Owing to the PBL in this case occurred in the skull, it is also need to be clinically and pathologically distinguished with some other diseases such as central nervous system metastases tumors, germ cell tumors, malignant melanoma, glioblastoma multiform and so on which also occurring in the brain. Central nervous system metastases usually oppear at the cerebrum and duramater places, especially the cinerea and white matter junction. CT scan showing a clear boundary, equal density or low density round lesions, with obvious peritumoral edema. MRI showed low signal on T1WI, high signal in T2WI, obvious peritumoral edema, with enhanced signals when enhancement. The metastases tumors in the brain are round or confluent, with clear border, gray or brown, and other organs within the body is generally able to find the primary tumor. The tumor cells can be distinguished with PBL by morphology and immunophenotype. Germ cell tumors are similar to PBL in morphology, which also have round or oval large cells and prominent nucleoli, with a background of small lymphocytes, but 90% of the central nervous system germ cell tumors involving patients under the age of 20, the peak age of 10–12 years old. It commonly occures in the pineal gland, the secondary place is saddle. The germ cell tumors immunophenotypes are different from PBL, the former can express PLAP and CD117, also express low molecular weight keratin, but do not express LCA, and plasma cells markers. The tumor cells of malignant melanoma in morphology could be alike with PBL, such as increased nucleus and nucleolus, with deeply stained and coarse chromatin, the mitosis and necrosis are easy to find. Immunohistochemistry staining showed malignant melanoma express S100, HMB-45 and Melan-A, but PBL don’t express these markers. Glioblastoma multiform is a rare glial tumors occur in children, adolescents and young people, commonly sites are frontal lobe and parietal lobe, the mass usually has clearly manifested boundaries Cell differentiation to obesity glioblastoma in morphology also can be similar to PBL, the nucleus looks like plasma cell-like, but can be found fence-like or polar arrangement tumor cells around the hyperplastic blood vessels and necrosis. The former express GFAP, S100 and other neurogenic protein and glial markers to make a confirm diagnosis.

About prognosis and predictive factors of PBL, the clinical course is very aggressive. Most patients are stage III or IV when they see a doctor. In addition, these disease is not sensitive to chemotherapeutics, and lack of standard chemotherapy regimen. Most of the patients dying in the first year after the diagnosis [8], primary central nervous system lymphomas (PCNSL) coexsited with HIV, the median srvival was 1.3 months [9].Those not primary central neurous system PBL but involved CNS, the median overall survival is about one month [10].

This case report also emphasizes the importance of clinical and radiological correlation in the diagnosis of this lethal disease.

## Consent

Written informed consent was obtained from the patient for publication of this Case Report and any accompanying images. A copy of the written consent is available for review by the Editor-in-Chief of this journal.

## Abbreviations

PBL: Plasmablastic lymphoma; DLBCL: Diffuse large B cell lymphoma Central; CNS: central nervous system; PCNSLs: Primary central nervous system lymphomas; MRI: Magnetic resonance imaging.

## Competing interests

The authors declare that they have no competing interests.

## Authors’ contributions

LY Z, HY L and LX G collected the data and drafted the manuscript. LY Z and LX G carried out the gross examination and final diagnosis. L L and YW T carried out the immunohistochemistry staining. ZQ L provided the imaging data and diagnosis, XH S and ZY L participated in the gene arrangement detection. All authors have read and approved the final manuscript.
